# Benefits and Risks of Clopidogrel vs. Aspirin Monotherapy after Recent Ischemic Stroke: A Systematic Review and Meta-Analysis

**DOI:** 10.1155/2019/1607181

**Published:** 2019-12-01

**Authors:** Maurizio Paciaroni, Birsen Ince, Bo Hu, Jiann-Shing Jeng, Kursad Kutluk, Liping Liu, Min Lou, Vladimir Parfenov, Ka Sing Lawrence Wong, Babak Zamani, Dara Paek, Jung Min Han, Michael del Aguila, Shalini Girotra

**Affiliations:** ^1^Stroke Unit and Division of Cardiovascular Medicine, University of Perugia, Santa Maria della Misericordia Hospital, Perugia, Italy; ^2^Department of Neurology, Division of Cerebrovascular Diseases, Cerrahpasa Medical Faculty, Istanbul University, Istanbul, Turkey; ^3^Union Hospital Affiliated to Tongji Medical College of Huazhong University of Science and Technology, Wuhan, China; ^4^Stroke Center and Department of Neurology, National Taiwan University Hospital, Taipei, Taiwan; ^5^Department of Neurology, Dokuz Eylul University, Izmir, Turkey; ^6^Department of Neurology and Stroke Center, Beijing Tiantan Hospital, Capital Medical University, Beijing, China; ^7^The Second Affiliated Hospital of Zhejiang University, School of Medicine, Hangzhou, China; ^8^Department of Nervous Diseases and Neurosurgery, Sechenov First Moscow State Medical University, Moscow, Russia; ^9^Department of Medicine and Therapeutics, The Chinese University of Hong Kong, Hong Kong; ^10^Iranian Stroke Society, Tehran, Iran; ^11^Doctor Evidence, Santa Monica, CA, USA; ^12^Sanofi, General Medicines and Emerging Markets, Singapore

## Abstract

**Aim:**

Though combination of clopidogrel added to aspirin has been compared to aspirin alone in patients with stroke or transient ischemic attack, limited data exists on the relative efficacy and safety between clopidogrel and aspirin monotherapy in patients with a recent ischemic stroke. We aimed to compare clopidogrel versus aspirin monotherapy in this population.

**Methods:**

PubMed, Embase, and CENTRAL databases were searched from inception to May 2018 to identify clinical trials and observational studies comparing clopidogrel versus aspirin for secondary prevention in patients with recent ischemic stroke within 12 months. Pooled effect estimates were calculated using a random effects model and were reported as risk ratios with 95% confidence intervals.

**Results:**

Five studies meeting eligibility criteria were included in the analysis. A total of 29,357 adult patients who had recent ischemic stroke received either clopidogrel (*n* = 14, 293) or aspirin (*n* = 15, 064) for secondary prevention. Pairwise meta-analysis showed a statistically significant risk reduction in the occurrence of major adverse cardiovascular and cerebrovascular events (risk ratio 0.72 [95% CI, 0.53–0.97]), any ischemic or hemorrhagic stroke (0.76 [0.58, 0.99), and recurrent ischemic stroke (0.72 [0.55, 0.94]) in patients who received clopidogrel versus aspirin. The risk of bleeding was also lower for clopidogrel versus aspirin (0.57 [0.45, 0.74]). There was no difference in the rate of all-cause mortality between the two groups.

**Conclusions:**

The analysis showed lower risks of major adverse cardiovascular or cerebrovascular events, recurrent stroke, and bleeding events for clopidogrel monotherapy compared to aspirin. These findings support clinical benefit for single antiplatelet therapy with clopidogrel over aspirin for secondary prevention in patients with recent ischemic stroke.

## 1. Introduction

Stroke is the second most common cause of death and the third most common cause of disability worldwide [1]. Approximately 795,000 people in the United States experience a stroke each year, of which 87% are ischemic strokes [2]. Additionally, approximately 20% of patients with a primary diagnosis of stroke have a second stroke within two years, accounting for 185,000 annual cases in the United States [2, 3]. Those with recurrent strokes have higher costs per patient and are more likely to experience poor outcomes as compared to patients with primary stroke [2, 4–6]. Therefore, secondary stroke prevention in patients with a history of ischemic stroke is critical in reducing the overall burden of stroke. It is estimated that nearly 80% of secondary strokes can be prevented with antiplatelet therapy when combined with lifestyle changes [2, 7].

Current guidelines from the American Heart Association and American Stroke Association recommend antiplatelet therapy with aspirin for patients with ischemic stroke [7, 8]. Other approved antiplatelet treatment options including clopidogrel, aspirin/dipyridamole, and ticlopidine have been shown to be safe and effective for secondary prevention in this population, however, the relative safety and effectiveness among the different antiplatelet agents has still not been clearly established [9–13]. Dual antiplatelet therapy with clopidogrel in combination with aspirin has been compared to aspirin in reducing recurrent stroke in patients with minor stroke (within 12–24 hours from onset) or transient ischemic attack (TIA) [14, 15], but data on the efficacy and safety of clopidogrel compared to aspirin as single antiplatelet agents exclusively in patients with recent ischemic stroke is limited. The aim of this study was to conduct a systematic review and meta-analysis to compare the efficacy and safety of clopidogrel versus aspirin used as a monotherapy for secondary prevention in patients with recent ischemic stroke. Findings from this comprehensive update on the available body of evidence will guide healthcare professionals and decision makers on the selection of optimal antiplatelet agent for preventative use in patients with recent ischemic stroke.

## 2. Methods

### 2.1. Data Sources and Searches

Literature searches were performed by a medical librarian (HT) in PubMed, Embase, and the Cochrane Central Register of Controlled Trials for studies published from inception to May 2018. Search strategies are provided in Supplemental [Supplementary-material supplementary-material-1]. Search results were exported to Digital Outcome Conversion (DOC™) Library Management System (LMS, version 2.0), and duplicates were removed (Doctor Evidence, Santa Monica, CA) [16]. To ensure that potentially relevant studies were not overlooked, reference lists from other reviews and meta-analyses on the current topic were searched by hand. The Preferred Reporting Items for Systematic review and Meta-Analysis protocols (PRISMA) guidelines were followed [17].

### 2.2. Study Selection

Medical librarians screened titles and abstracts based on a standardized review protocol that defined study eligibility criteria using the PICOTSS format, which outlines the participants, interventions, comparators, outcomes, timing, setting, and study designs of interest (Table 1). Eligible studies were those that compared the beneficial and harmful effects of clopidogrel and aspirin monotherapies for the prevention of recurrent stroke and other cardiovascular complications in patients who experienced ischemic stroke in the previous year. Studies that also enrolled patients with TIA were included only if data for ischemic stroke patients were reported separately. Randomized controlled trials (RCT) and comparative observational studies with at least 1-month follow-up were included. There was no restriction for study setting. Studies were required to report at least one outcome of interest.

Efficacy outcomes included recurrent stroke of any type, recurrent ischemic stroke, and all-cause mortality. Also collected were major adverse cardiovascular and cerebrovascular events (MACCE). MACCE was defined as a composite outcome that included two or more of the following: recurrent stroke, myocardial infarction, unstable angina, coronary revascularization, aortic aneurysm rupture, peripheral artery disease, vascular death, and sudden death. Safety outcomes included any reported bleeding events, including intracranial hemorrhage and gastrointestinal bleeding. Only studies published in English were included for review of the full text. Studies presented only in conference abstracts without an associated publication were excluded, as this data is not peer reviewed and there is often limited information available on the details of the study and patient characteristics. A PRISMA flow diagram was created based on the search results and study selection.

### 2.3. Data Extraction and Quality Assessment

Relevant information was extracted by two independent reviewers using the Doctor Evidence software platform version 2.0 [16]. The following information was collected: study design, study location, publication year, number of patients in each arm, intervention, comorbidities, outcomes of interest, and study inclusion/exclusion criteria. When available, the definitions and descriptions of stroke and outcomes provided by the authors were also captured. Any discrepancies in data extraction were resolved by discussion. All terms (characteristics and outcomes) were collected as reported by study authors and synonyms were “bound” before analysis using the DOC™ Ontology System. Detailed methods are described elsewhere [16].

Quality assessment of the included studies was performed by two independent reviewers using the Cochrane Collaboration tool for assessing the risk of bias for randomized trials and the Newcastle-Ottawa Scale for cohort studies [18, 19].

### 2.4. Statistical Analysis

Pairwise meta-analysis for outcomes were performed using the inverse-variance weighted random effects model based on the DerSimonian and Laird 1986 method to estimate the risk ratio (RR) and 95% confidence interval (CI) [20]. A random effects model took into account both within-study and between-study variability. Heterogeneity was assessed using the *I*-squared (*I*^2^) statistic, which describes the percentage of variation across the studies that is due to heterogeneity rather than chance [21]. Percentages of approximately 25%, 50%, and 75% were considered to have low, moderate, or high heterogeneity, respectively. Patient and study characteristics were visually assessed for any potential heterogeneity across studies that might have affected pooled effect estimates. Publication bias could not be assessed due to the limited number of studies available for all outcomes. Multiple analyses were also conducted to consider varying definitions for the composite MACCE outcome in the event that studies reported more than one composite outcome that could qualify as MACCE. If a study reported multiple composite vascular outcomes, the most inclusive composite outcome was selected for the main analysis [22]. All analyses were performed on DOC™ Data, using R (metaphor package [v.2.0.0]) [23].

## 3. Results

### 3.1. Literature Search

**Figure 1:**
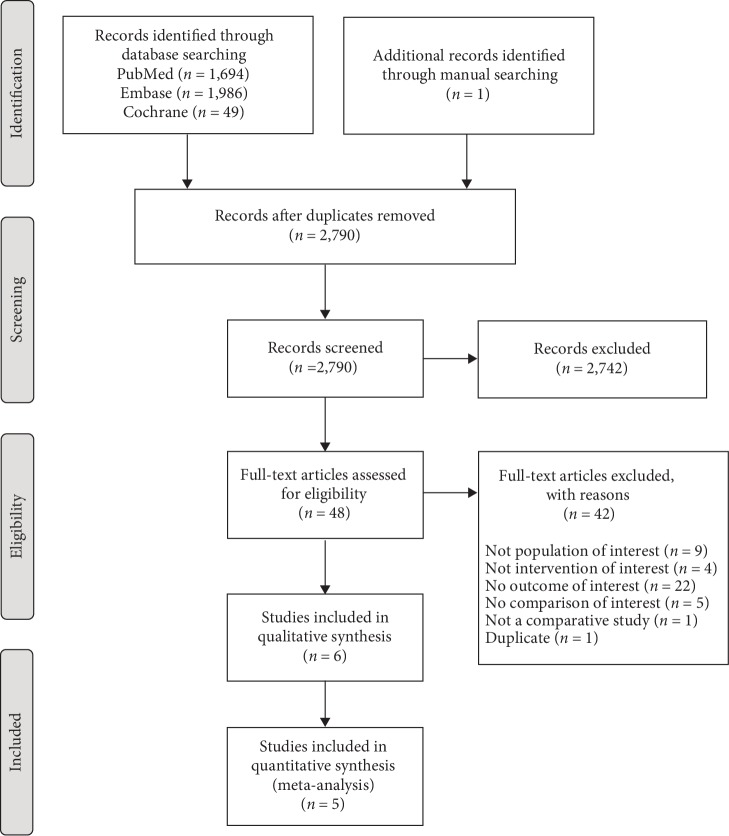
PRISMA flow diagram showing study identification and selection.

Of the 2,790 records identified in our search, 2,742 were excluded through title and abstract screening. One additional study was identified manually after the initial search [24]. Among the 48 full texts reviewed, six studies met eligibility criteria (Figure 1). One paper [21] reported on diabetic patients for the same retrospective cohort [22] and was therefore excluded, resulting in a total of five studies included in the meta-analysis.

### 3.2. Quality Assessment

**Figure 2:**
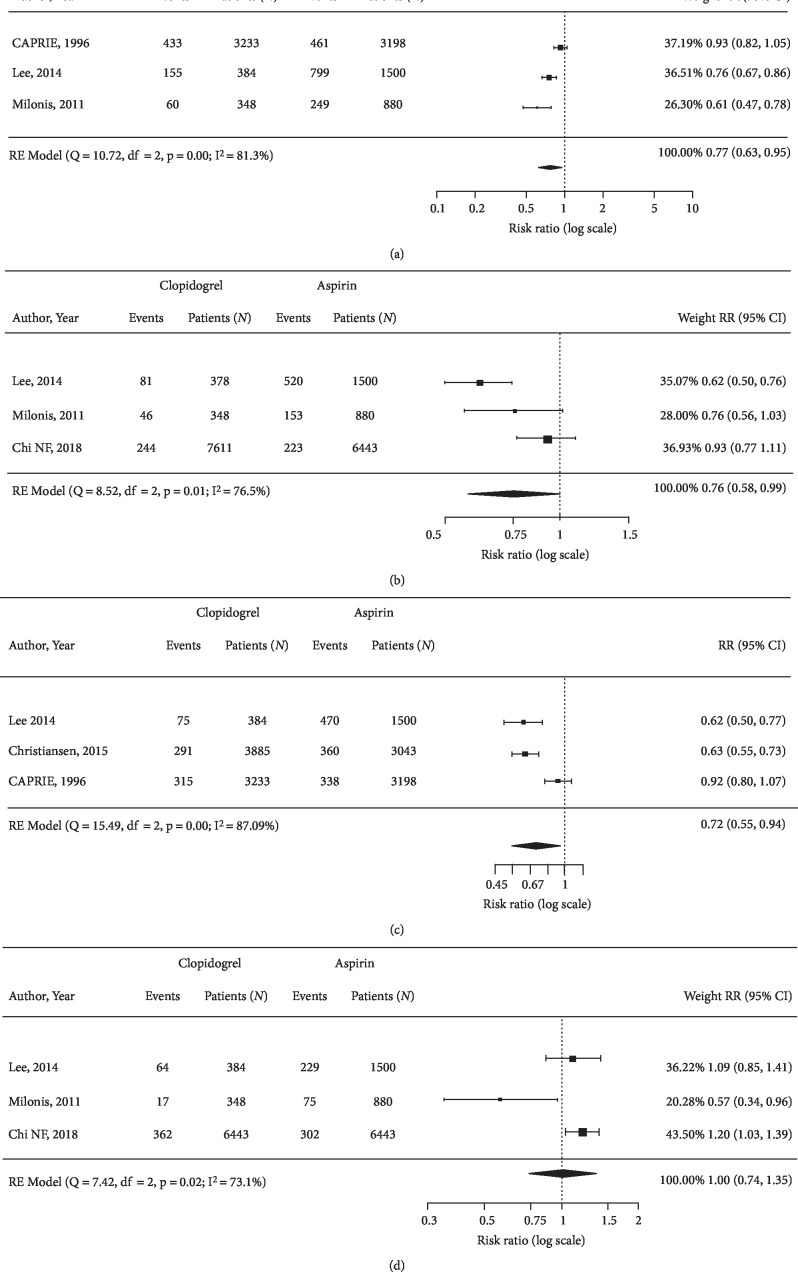
Forest plots showing pooled risk ratio of (a) MACCE, (b) any ischemic or hemorrhagic stroke, (c) recurrent ischemic stroke, and (d) all-cause mortality.

**Figure 3:**
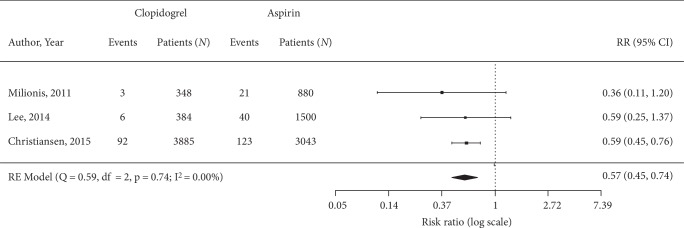
Forest plot showing pooled risk ratio for bleeding events.

The quality assessment for the included studies is presented in Supplemental [Supplementary-material supplementary-material-1]. The risk of bias was rated as low for all seven domains for the RCT. All of the observational studies were rated as of high quality, with Newcastle Ottawa Scale scores of eight or nine. The *I*^2^ statistic for each outcome is shown in Figures 2 and 3.

### 3.3. Study and Patient Characteristics

One RCT and four retrospective cohort studies met the PICO criteria for inclusion. Study and patient characteristics of the studies are shown in Table 2. All retrospective cohort studies were conducted in a single country (Denmark, Greece, and Taiwan), whereas the RCT was conducted across 16 countries. All studies were published in peer-reviewed journals.

A total of 29,357 adult patients who had recent ischemic stroke received either clopidogrel (*n* = 14, 293) or aspirin (*n* = 15, 064) for secondary prevention. The proportion of males ranged from 48% to 73%. The mean age ranged from 64.5 years to 77.6 years. The length of study follow-up ranged from one year to five years. Comorbidities such as diabetes, hypertension, hyperlipidemia, coronary artery disease, myocardial infarction, congestive heart failure, and peripheral artery disease were prevalent at baseline (Table 2). There was no observed trend in different patient characteristics between two treatment groups upon inspection. Two studies did not enroll patients with a history of atrial fibrillation [22, 25] and three studies did not enroll patients who had received anticoagulation therapy [24–26]. The average daily dose of clopidogrel was similar across the studies that reported the average daily dose (~75 mg/day), whereas the average daily dosage of aspirin varied from 102 mg/day to 325 mg/day. The reported outcome definitions varied across studies, most notably for MACCE and bleeding events (Supplemental [Supplementary-material supplementary-material-1]).

### 3.4. Comparative Efficacy and Effectiveness

Results of the pairwise meta-analysis showed a statistically significantly lower risk of MACCE among patients who received clopidogrel compared to those who received aspirin (RR 0.77 [95% CI, 0.63, 0.95]; Figure 2(a)). The risks of stroke of any type, ischemic or hemorrhagic, (0.76 [0.58, 0.99]; Figure 2(b)), and recurrent ischemic stroke (0.72 [0.55, 0.94]; Figure 2(c)) were statistically significantly lower with clopidogrel therapy. There was no difference found for the rate of all-cause mortality (Figure 2(d)).

Sensitivity analysis using a more restrictive definition for MACCE reported in Lee et al. 2014 (i.e., ischemic or hemorrhagic stroke or MI) showed a similar result; patients receiving clopidogrel experienced a lower rate of MACCE (0.72 [0.47, 0.78]) compared to aspirin (Supplemental [Supplementary-material supplementary-material-1] and Supplemental [Supplementary-material supplementary-material-1]).

### 3.5. Safety

Bleeding events were reported in three studies (Supplemental [Supplementary-material supplementary-material-1]). Statistically significant reduction in risk of bleeding events was shown for clopidogrel (0.57 [0.45, 0.74]) compared to aspirin (Figure 3).

## 4. Discussion

The results of our review suggest clinical benefit for single antiplatelet therapy with clopidogrel over aspirin in recent ischemic stroke patients. Pooled relative risk estimates for major composite cardiovascular and cerebrovascular events, recurrence of ischemic stroke, or any ischemic or hemorrhagic stroke were all significantly lower for clopidogrel monotherapy compared to aspirin. Risk of bleeding events were also significantly lower with clopidogrel therapy.

Although broad searches were conducted to identify and include all available literature and sensitivity analyses were run to test the robustness of our findings, there are some limitations to be considered when interpreting these results. Studies often reported composite outcomes as their primary outcome because a smaller sample size is required to adequately power a composite outcome as compared to individual outcomes. Definitions of MACCE and recurrent stroke that most closely resembled the definitions reported in other studies were used, but data collection was limited to the published study-level results. The between-study heterogeneity found in our analyses remain unexplained due to the nature of observational studies. Thus, the pooled preventative effects of clopidogrel over aspirin shown for MACCE and recurrent stroke may underestimate the true preventative effects of clopidogrel.

Bleeding event data was only available from retrospective cohort studies. Any reported bleeding events including both composite bleeding events and specific bleeding events were combined (e.g., intracranial hemorrhage and gastrointestinal bleeding). These bleeding events were captured from insurance databases or national registries, and it is reasonable to assume that these events were severe enough to require medical attention (e.g., office visit or hospitalization). However, due to the nature of the claim-based database and national registries, we were unable to compare bleeding events by severity. In one study [22], the authors noted that clopidogrel was prescribed only for those with pre-existing gastrointestinal ulcers or bleeding issues or for those who have already failed on aspirin. This is important to note as the results may not reflect the true rate of bleeding events associated with clopidogrel use.

Selecting optimal therapy for secondary stroke prevention requires careful attention, as these patients often present with comorbidities and other risk factors which may influence prescription and treatment effectiveness. The boxed label warning for clopidogrel cautions against use of clopidogrel in patients with impaired platelet reactivity due to known genetic polymorphisms of CYP2C19 [28]. The majority of studies in this review included data from before 2010 and genotype testing or platelet monitoring via platelet function tests may not have been performed, as routine testing is still not included in any current guideline recommendations. The included observational studies used claim-based or registry data, and in such real-world settings, the selection of antiplatelets was based on physicians' preference and receipt of clopidogrel often depended on insurance or drug formularies and requirements by country. However, this information was not reported in the studies. Due to the nature of the retrospective cohort studies included in this analysis, clinicians should be aware that unknown and therefore unmeasured confounders might have affected our effect estimates differentially.

Aspirin remains the recommended antiplatelet therapy for patients with ischemic stroke in current guidelines [8]. Published trial data suggests clopidogrel as single antiplatelet therapy is safe and effective for secondary prevention compared to aspirin and the combination of aspirin/dipyridamole [27, 29]. However, the strength of the evidence in support of clopidogrel over other antiplatelet agents is limited by the few numbers of studies that make direct comparison to clopidogrel as single antiplatelet therapy, and more recent data is based on the combination of clopidogrel and aspirin in mixed populations with ischemic stroke or TIA. In the absence of further clinical trials, indirect evidence obtained through further meta-analysis and data from prospective patient registries may provide valuable insights on the efficacy and safety of clopidogrel relative to aspirin for secondary prevention patients with ischemic stroke.

To our knowledge, this is the first systematic review and meta-analysis conducted in patients with recent ischemic stroke specifically, as most of the current evidence is based on stroke and TIA populations. We included all published clinical trials and observational studies that made direct comparison of clopidogrel and aspirin monotherapy for secondary prevention in patients with ischemic stroke. Data from observational studies were included to explore the benefits and harms for this population in the real-world setting. Because there is limited data on the relative efficacy and safety of clopidogrel compared to aspirin alone, these findings can add valuable information to help clinicians and policymakers in selection of antiplatelet therapy for secondary prevention following recent ischemic stroke.

## 5. Conclusions

This systematic review and meta-analysis found that clopidogrel monotherapy was associated with significantly lower risks of MACCE, recurrent stroke, and bleeding events compared to aspirin in patients with ischemic stroke. The results of the analysis support clinical benefit for single antiplatelet therapy with clopidogrel over aspirin for secondary prevention in patients with recent ischemic stroke. There were few studies included in this review and data were based largely on retrospective observational data. More longitudinal data and high-quality studies are warranted to verify the findings of this systematic literature review and meta-analysis.

## Figures and Tables

**Table 1:** Study inclusion and exclusion criteria in PICOTSS format.

Population	(i) Patients with recent ischemic stroke within the previous year
	(ii) Subgroup data for ischemic stroke patients in studies with mixed stroke/TIA populations
Interventions	Clopidogrel monotherapy (any dosage) for at least four weeks
Comparators	Aspirin monotherapy (any dosage) for at least four weeks
Outcomes	*Efficacy outcomes*
	(i) MACCE
	(a) Defined as any composite outcomes that included two or more of the following: recurrent stroke, myocardial infarction, unstable angina, coronary revascularization, aortic aneurysm rupture, peripheral artery disease, vascular death and sudden death
	(ii) Recurrent stroke (ischemic and hemorrhagic)
	(iii) Recurrent ischemic stroke
	(iv) Mortality
	*Safety outcome*
	(i) Bleeding risk
	(a) Intracranial
	(b) Gastrointestinal
	(c) Any reported
Timing	Minimum study duration/follow-up of at least four weeks (one month)
Setting	No restriction
Study design	(i) Randomized controlled trials
	(ii) Comparative observational studies

MACCE: major adverse cardiovascular and cerebrovascular events; TIA: transient ischemic attack.

**Table 2:** Study and patient characteristics of the included studies.

Study, design, location	Inclusion criteria	Exclusion criteria	Follow-up duration	Treatment groups (*N*)	Age (yr)^*^	Male, %	Comorbidities, %
CAPRIE (1996) [27]^†^, RCT, 16 countries	Patients (≥21 years) with ischemic stroke (retinal and lacunar infarction) with the following:	(i) Severe cerebral deficit likely to lead to patient being bedridden or demented	Minimum of 1 year and maximum of 3 year	Clopidogrel (3,233) 75 mg/d	64.7 ± 11.0	63	Angina: 17
	(i) focal neurological deficit likely to be of atherothrombotic origin	(ii) Carotid endarterectomy after qualifying stroke	Mean follow-up: 1.91 years				Atrial fibrillation: 4
	(ii) onset ≥ 1 week and ≤ 6 months before randomization	(iii) Qualifying stroke induced by carotid endarterectomy or angiography	Total person-time: Clopidogrel: 6,054 person-years at risk;				Cardiomegaly: 5
	(iii) neurological signs persisting ≥1 week from stroke onset	(iv) Unlikely to be discharged alive after qualifying event	Aspirin: 5,979 person-years at risk				CHF: 4
	(iv) CT or MRI ruling out hemorrhage or non-relevant disease	(v) Severe co-morbidity likely to limit patient's life expectancy to <3 years					Diabetes: 26
		(vi) Uncontrolled hypertension					Hyperlipidemia: 38
							Hypertension: 65
							Ischemic stroke: 19
							Myocardial infarction: 11
							TIA/RIND: 19
				Aspirin (3,198) 325 mg/d	64.5 ± 11.2	64	Angina: 17
							Atrial fibrillation: 4
							Cardiomegaly: 6
							CHF: 4
							Diabetes: 25
							Hyperlipidemia: 37
							Hypertension: 65
							Ischemic stroke: 17
							Myocardial infarction: 13
							TIA/RIND: 19
Chi et al. (2018) [24], Retrospective cohort, Taiwan	Adult patients from the Taiwanese Stroke Registry who had ischemic stroke and whose survival statuses one year after the index stroke were confirmed	(i) Received a combination of aspirin and clopidogrel,	For 1 year after the diagnosis of ischemic stroke	Clopidogrel (6,443)	71.4 ± 13.2	60.5	Atrial fibrillation: 4.3
		(ii) Received other medicine including Aggrenox, ticlopidine, cilostazol, or warfarin					CHD: 2.20
							CVA/TIA: 32.7
							Diabetes: 42.1
							Heart disease: 34.4
		(iii) Died during hospitalization for acute ischemic stroke					Hyperlipidemia: 45.9
		(iv) With missing data					Hypertension: 78.6
		(v) Died at discharge					IHD: 17.1
		(vi) Had recurrent stroke before discharge		Aspirin (6,443)			Myocardial infarction: 0.34
					71.8 ± 16.3	60.2	Atrial fibrillation: 4.33
							CHD: 2.20
							CVA/TIA: 34.6
							Diabetes: 42.3
							Heart disease: 33.0
							Hyperlipidemia: 45.0
							Hypertension: 79.2
							IHD: 17.9
							Myocardial infarction: 0.25
Christiansen et al. (2015) [25], Retrospective cohort, Denmark	Patients with first-time ischemic stroke discharged from Jan. 2017 to Dec. 2010 and those who survived the first 30 days after stroke	Atrial fibrillation or anticoagulation therapy before or up to 30 days after discharge	From 30 days after discharge until patients had an outcome, died, emigrated, or 1 year after discharge, whichever comes first	Clopidogrel (3,885)	68.6 (59.2–77.6)	49	Diabetes: 11.7
							Bleeding: 8.2
			Median follow-up: 335 days [335–335]				Cancer: 6.3
			Total person-time: Clopidogrel: 3,364 person-years; Aspirin: 2,475 person-years				COPD: 7.1
							Heart failure: 5.9
							Hypertension: 42.3
							Myocardial infarction: 13.5
				Aspirin (3,043)			PAD: 4.6
					75.3 (64.5–83.7)	48	Bleeding: 13.3
							Cancer: 6.8
							COPD: 8.4
							Diabetes: 12.5
							Heart failure: 7.3
							Hypertension: 43.2
							Myocardial infarction: 11
							PAD: 4.1
Lee et al. (2014) [22], Retrospective cohort, Taiwan	Hospitalized adults who were admitted with a primary diagnosis of ischemic stroke (index stroke) between 2003 and 2009 and received continuous aspirin treatment ≥30 days before the index stroke	(i) Atrial fibrillation, valvular heart disease, or coagulopathy	Mean follow up: 2.4 years	Clopidogrel^‡^ (384)	70.8 ± 9.5	60	Diabetes: 44.0
		(ii) Those with poor drug adherence (medication possession ratio ≤80%)		Average daily dose: 74.6 mg			GI bleeding/peptic ulcer: 18.8
							Hyperlipidemia: 20.3
							Hypertension: 57.3
							IHD: 16.7
				Aspirin (1,500)			Stroke/TIA: 22.7
				Average daily dose: 101.9 mg	71.1 ± 10.2	60	Diabetes: 49.1
							GI bleeding/peptic ulcer: 2.6
							Hyperlipidemia: 21.8
							Hypertension: 52.0
							IHD: 18.9
							Stroke/TIA: 18.7
Milionis et al. (2011) [26], Retrospective cohort, Greece	Patients who were hospitalized due to an acute ischemic stroke (atherothrombotic, lacunar, cryptogenic) and had an indication to receive antiplatelet therapy	Those who were treated with coumadin	For 5 years from index stroke	Clopidogrel (348) Average daily dose: 75 mg/d	77.6 ± 11.0	73	CAD:18.4
			Mean follow-up: Clopidogrel: 38.5 ± 20.4 months;				Diabetes: 66.1
			Aspirin: 40.9 ± 22.2 months				Hyperlipidemia: 46.3
							Hypertension: 31.3
							PAD: 6.1
				Aspirin (880)			TIA: 14.1
				Average daily dose: 104 mg/d	67.6 ± 11.8	70	CAD: 18.9
							Diabetes: 71.4
							Hyperlipidemia: 38.4
							Hypertension: 29.5
							PAD: 5.5
							TIA: 14.7

^*^Age presented as mean age ± standard deviation or median age with interquartile range. ^†^Only subgroup of patients with ischemic stroke at baseline are presented. ^‡^The Taiwan National Health Insurance Bureau provided reimbursement for the use of clopidogrel in patients with ischemic stroke who are allergic to aspirin, have peptic ulcer, or aspirin treatment failure. CAD: coronary artery disease; CHF: congestive heart failure; COPD: chronic obstructive pulmonary disease; CT: computed tomography; CVA: cerebrovascular attack; IHD: ischemic heart disease; MRI: magnetic resonance imaging; PAD: peripheral artery disease; RCT: randomized controlled trial; RIND: reversible ischemic neurological deficit; TIA: transient ischemic attack.

## Supplementary Materials

Supplementary Materials The supplementary PDF includes summaries of the search strings, in addition to further information on methodology and results.
