# Supplementary material for: Benefits and Risks of Clopidogrel vs. Aspirin Monotherapy after Recent Ischemic Stroke: A Systematic Review and Meta-Analysis
Source: Cardiovasc Ther. 2019 Dec 1;2019:1607181. doi: 10.1155/2019/1607181 (PMC6913341; doi:10.1155/2019/1607181)
Supplement: Supplementary Materials — The supplementary PDF includes summaries of the search strings, in addition to further information on methodology and results. [file 1607181.f1.pdf]

SUPPLEMENTAL MATERIAL

Benefits and Risks of Clopidogrel vs. Aspirin Monotherapy After Recent Ischemic Stroke: A Systematic Review and Meta-analysis

List of Abbreviations

CAD	Coronary artery disease
CHF	Congestive heart failure
COPD	Chronic obstructive pulmonary disease
CT	Computed tomography
DOC	Digital outcome conversion
ERC	Evidence review committees
HF	Heart failure
HTN	Hypertension
IHD	Ischemic heart disease
LMS	Library management system
MACCE	Major adverse cardiovascular and cerebrovascular events
MI	Myocardial infraction
MRI	Magnetic resonance imaging
PAD	Peripheral artery disease
PICOTSS	Participant, intervention, comparator, outcome, timing, setting, and study design
PRISMA	Preferred Reporting Items for Systematic Reviews and Meta-Analyses
RCT	Randomized controlled trial
RIND	Reversible ischemic neurological deficit
TIA	Transient ischemic attack

Supplemental Tables

Database: PubMed		
**#**	**Search date: 25 May 2018**	
1	(stroke[Mesh] OR stroke[tiab] OR strokes[tiab] OR cerebrovascular accident*[tiab] OR poststroke*[tiab] OR post-stroke[tiab] OR apoplex*[tiab] OR brain accident*[tiab] OR brain vascular accident[tiab] OR brain ischemic attack[tiab] OR "brain ischaemic attack"[tiab])	Stroke terms
2	("clopidogrel" [Supplementary Concept] OR clopidogrel [tiab] OR "SC 25989C" [tiab] OR SC25989C [tiab] OR SC-25989C [tiab] OR SC25990C [tiab] OR SC-25990 [tiab] OR SR-25989 [tiab] OR SR25989 [tiab] OR PCR4099 [tiab] OR PCR-4099 [tiab] OR iscover [tiab] OR plavix [tiab])	Clopidogrel terms
3	("aspirin"[mesh] OR aspirin[tiab] OR Acetylsalicylic Acid[tiab] OR Acetysal[tiab] OR Acuprin[tiab] OR Acylpyrin[tiab] OR Aloxiprimum[tiab] OR Anacin[tiab] OR Ascriptin[tiab] OR Aspergum[tiab] OR Aspidrox[tiab] OR Aspir-Mox[tiab] OR Aspirtab[tiab] OR Aspir-trin[tiab] OR Bufferin[tiab] OR Buffex[tiab] OR Colfarit[tiab] OR Dispril[tiab] OR Easprin[tiab] OR Easprin[tiab] OR Easprin[tiab] OR Ecotrin[tiab] OR Empirin[tiab] OR Endosprin[tiab] OR Entaprin[tiab] OR Entercote[tiab] OR Fasprin[tiab] OR Genacote[tiab] OR Gennin-FC[tiab] OR Genprin[tiab] OR Halfprin[tiab] OR Magnaprin[tiab] OR Magnecyl[tiab] OR Micristin[tiab] OR Miniprin[tiab] OR Minitabs[tiab] OR Polopirin[tiab] OR Polopiryna[tiab] OR Ridiprin[tiab] OR Sloprin[tiab] OR Solprin[tiab] OR Solupsan[tiab] OR Uni-Buff[tiab] OR Uni-Tren[tiab] OR Valomag[tiab] OR Zorprin[tiab])	Aspirin terms
4	2 AND 3	Clopidogrel OR Aspirin
5	1 AND 4	Population AND Intervention
6	(animals[mh] NOT humans[mh])	Humans only
7	5 NOT 6	
8	ENG [la]	Remove non-clinical studies
9	8 AND 9	
10	(stroke[Mesh] OR stroke[tiab] OR strokes[tiab] OR cerebrovascular accident*[tiab] OR poststroke*[tiab] OR post-stroke[tiab] OR apoplex*[tiab] OR brain accident*[tiab] OR brain vascular accident[tiab] OR brain ischemic attack[tiab] OR "brain ischaemic attack"[tiab])	English language
11	("clopidogrel" [Supplementary Concept] OR clopidogrel [tiab] OR "SC 25989C" [tiab] OR SC25989C [tiab] OR SC-25989C [tiab] OR SC25990C [tiab] OR SC-25990 [tiab] OR SR-25989	1,694 results

	[tiab] OR SR25989 [tiab] OR PCR4099 [tiab] OR PCR-4099 [tiab] OR iscover [tiab] OR plavix [tiab])	
**Database: Embase (via Ovid)**		
**#**	**Search date: 25 May 2018**<br>**Segment used: 1974 to 2018 May 23**	
1	*cerebrovascular accident/ or (stroke or strokes or cerebrovascular accident$ or poststroke$ or brain ischemic attack or brain ischaemic attack).ti,ab.	Stroke terms
2	*clopidogrel/ or (clopidogrel or SC-25989C or SC25989C or SC25990C or SC-25990C or SR-25989 or SR25989 or PCR 4099 or PCR-4099 or iscover or plavix).ti,ab.	Clopidogrel terms
3	*aspirin/ or (aspirin or Acetylsalicylic Acid or Acetysal or Acuprin or Acylpyrin or Aloxiprimum or Anacin or Ascriptin or Aspergum or Aspidrox or Aspir-Mox or Aspirtab or Aspir-trin or Bufferin or Buffex or Colfarit or Dispril or Easprin or Easprin or Ecotrin or Empirin or Endosprin or Entaprin or Entercote or Fasprin or Genacote or Gennin-FC or Genprin or Halfprin or Magnaprin or Magnecyl or Micristin or Miniprin or Minitabs or Polopirin or Polopiryna or Ridiprin or Sloprin or Solprin or Solupsan or Uni-Buff or Uni-Tren or Valomag or Zorprin).ti,ab.	Aspirin terms
4	2 and 3	
5	1 and 4	Population AND interventions
6	5 not ((exp animal/ or nonhuman/) not exp human/)	Humans only
7	limit 6 to english language	English language
8	7 not (case report$.ti. or *in vitro study/)	
9	8 not (book or book series or conference abstract or conference proceeding or "conference review" or editorial or letter or note or "review" or book review or chapter or patent).pt.	Remove non-clinical, in vitro, and case reports
10	limit 8 to (yr="2012 -Current" and conference abstract)	Limit conference abstracts to 2012
11	remove duplicates from 9	
12	remove duplicates from 10	
13	11 or 12	1,986 results
**Database: Cochrane Central Register of Controlled Trials (CENTRAL)**		
**#**	**Search date: 25 May 2018**	
1	stroke or strokes or cerebrovascular accident* or poststroke* or brain ischemic attack or brain ischaemic attack:ti,ab,kw (Word variations have been searched)	Stroke terms
2	MeSH descriptor: [Stroke] explode all trees	
3	1 or 2	
4	clopidogrel or SC-25989C or SC25989C or SC25990C or SC-25990C or SR-25989 or SR25989 or PCR 4099 or PCR-4099 or iscover or plavix:ti,ab,kw (Word variations have been searched)	Clopidogrel terms
5	MeSH descriptor: [Aspirin] explode all trees	

6	aspirin or Acetylsalicylic Acid or Acetysal or Acuprin or Acylpyrin or Aloxiprimum or Anacin or Ascriptin or Aspergum or Aspidrox or Aspir-Mox or Aspirtab or Aspir-trin or Bufferin or Buffex or Colfarit or Dispril or Easprin or Easprin or Ecotrin or Empirin or Endosprin or Entaprin or Entercote or Fasprin or Genacote or Gennin-FC or Genprin or Halfprin or Magnaprin or Magnecyl or Micristin or Miniprin or Minitabs or Polopirin or Polopiryna or Ridiprin or Sloprin or Solprin or Solupsan or Uni-Buff or Uni-Tren or Valomag or Zorprin:ti,ab,kw (Word variations have been searched)	Aspirin terms
7	5 or 6	
8	3 and 4 and 7	Population AND interventions
9	8 not (pubmed or embase):an	49 results

Supplemental Table I. Search strategies for PubMed, Embase, and the Cochrane Central Register of Controlled Trials (CENTRAL)

**Randomized controlled trial**[*]							
**Study**	**Random sequence generation**	**Allocation concealment**	**Blinding of participants and personnel**	**Blinding of outcome assessment**	**Incomplete outcome data**	**Selective reporting**	**Other bias**
CAPRIE (1996)[1]	Low risk	Low risk	Low risk	Low risk	Low risk	Low risk	Low risk

**Retrospective cohort studies**[†]					
**Study**	**Study design**	**Selection**	**Comparability**	**Outcome**	**Final score**
Chi et al. (2018)[2]	Cohort	****	**	***	9
Christiansen et al. (2015)[3]	Cohort	****	**	***	9
Lee et al. (2014)[4]	Cohort	***	**	***	8
Milionis et al. (2011)[5]	Cohort	***	**	***	8

Supplemental Table II. Quality assessment for the included studies

[*] The Cochrane risk-of-bias tool for randomized trials was used. [†] The Newcastle Ottawa Scale for cohort studies were used.

Study	Definitions
**Major adverse cardiovascular and cerebrovascular events (MACCE)**	
CAPRIE (1996)[1*]	Includes ischemic stroke, MI, or vascular death
Lee et al. (2014)[4]	Composite of any stroke (ischemic or hemorrhagic), or MI
	Composite of any stroke (ischemic or hemorrhagic), MI, or all-cause mortality
Milionis et al. (2011)[5]	Includes stroke recurrence, MI, unstable angina, coronary revascularization, aortic aneurysm rupture, peripheral atherosclerotic artery diseases, and sudden death
**All recurrent stroke**	
Lee et al. (2014)[4]	Ischemic or hemorrhagic
Milionis et al. (2011)[5]	A cerebrovascular event of sudden onset lasting for ≥24 h subsequent to the initial stroke that clearly resulted in a new or an increase in an existing neurological deficit
Chi et al. (2018)[2]	As reported in the Taiwanese Stroke Registry
**Recurrent ischemic stroke**	
CAPRIE (1996)[1]	Ischemic stroke, non-fatal: acute neurological vascular event with focal signs for ≥ 24 h if in a new location, without evidence of intracranial hemorrhage/ If worsening of previous event, must have lasted >1 week, or more than 24 h if accompanied by appropriate CT or MRI findings
	Ischemic stroke, fatal: either death within 28 days after the onset of signs or symptoms of the acute ischemic stroke (described above), in the absence of other clear causes, or on necropsy finding
Christiansen et al. (2015)[3]	NR
Lee et al. (2014)[4]	NR
**All-cause mortality**	
Chi et al. (2018)[2]	As reported in the Taiwanese Stroke Registry
Lee et al. (2014)[4]	NR
Milionis et al. (2011)[5]	Sudden death
**Bleeding events**	
Christiansen et al. (2015)[3]	Gastrointestinal bleeding, intracranial hemorrhage, or bleeding from the urogenital or respiratory system
Lee et al. (2014)[4]	Intracranial hemorrhage
Milionis et al. (2011)[5]	Bleeding, severe: Includes peptic ulcer disease-related hemorrhage, which required blood transfusion or hospitalization and intracranial bleeding

Supplemental Table III. Relevant outcomes and definitions provided from the included studies
NR: not reported; MI: myocardial infarction; CT: computed tomography; MRI: magnetic resonance imaging.

[*] For CAPRIE (1996), only outcomes reported for the subgroup of patients with ischemic stroke at baseline are presented.

Study	Treatment group (N)	Patients, n (%)	Event rates	Definitions
**Major adverse cardiovascular and cerebrovascular events (MACCE)**				
CAPRIE (1996)[1*]	Clopidogrel (3,233)	433 (13.39)	7.15% per person-year	Ischemic stroke, MI, or vascular death
	Aspirin (3,198)	461 (14.42)	7.71% per person-year	
Lee et al. (2014)[4]	Clopidogrel (384)	155 (40.4)		Any stroke (ischemic or hemorrhagic), MI, or all-cause mortality
	Aspirin (1,500)	799 (53.3)		
	Clopidogrel (384)	91 (23.7)	9.9% per person-year	Any stroke (ischemic or hemorrhagic) or MI
	Aspirin (1,500)	570 (38.0)	15.8% per person-year	
Milionis et al. (2011)[5]	Clopidogrel (348)	60 (17.2)		Stroke recurrence, MI, unstable angina, coronary revascularization, aortic aneurysm rupture, peripheral atherosclerotic artery diseases, and sudden death
	Aspirin (880)	249 (28.3)		
**All recurrent stroke**				
Chi et al. (2018)[2]	Clopidogrel (6,443)	244 (3.79)		
	Aspirin (6,443)	223 (34.6)		
Lee et al. (2014)[4]	Clopidogrel (384)	81 (21.2)		
	Aspirin (1,500)	520 (34.7)		
Milionis et al. (2011)[5]	Clopidogrel (348)	46 (13.2)		
	Aspirin (880)	153 (17.4)		
**Recurrent ischemic stroke**				
CAPRIE (1996)[1*]	Clopidogrel (3,233)	315 (9.7)		Fatal and non-fatal
	Aspirin (3,198)	338 (10.6)		
	Clopidogrel (3,233)	17 (0.5)		Fatal
	Aspirin (3,198)	16 (0.5)		
	Clopidogrel (3,233)	298 (9.2)		Non-fatal

	Aspirin (3,198)	322 (10.1)		
Christiansen et al. (2015)[3†]	Clopidogrel (3,885)	291 (7.5)	0.09 events per person-year	
	Aspirin (3,043)	360 (11.8)	0.15 events per person- year	
Lee et al. (2014)[4]	Clopidogrel (384)	75 (19.5)		
	Aspirin (1,500)	470 (31.3)		
**All-cause mortality**				
Chi et al. (2018)[2]	Clopidogrel (6,443)	362 (5.62)		
	Aspirin (6,443)	302 (4.89)		
Lee et al. (2014)[4]	Clopidogrel (384)	64 (16.7)		
	Aspirin (1,500)	229 (15.3)		
Milionis et al. (2011)[5]	Clopidogrel (348)	17 (5.0)		
	Aspirin (880)	75 (9.0)		

Supplemental Table IV. Efficacy outcomes reported in the included studies

[*] Follow-up period: 6,054 person-years for clopidogrel and 5,979 person-years for aspirin.

† Follow-up period: 3,364 person-years for clopidogrel and 2,475 person-years for aspirin.

Study	Treatment group (N)	Patients, n (%)	Definitions
Christiansen et al. (2015)[3*]	Clopidogrel (3,885)	92 (2.4)	Gastrointestinal bleeding, intracranial hemorrhage, or bleeding from the urogenital or respiratory system
	Aspirin (3,043)	123 (4.0)	
Lee et al. (2014)[4†]	Clopidogrel (384)	6 (1.6)	Intracranial hemorrhage
	Aspirin (1,500)	50 (3.3)	
Milionis et al. (2011)[5‡]	Clopidogrel (348)	3 (0.9)	Peptic ulcer disease-related hemorrhage, which required blood transfusion or hospitalization and intracranial bleeding
	Aspirin (880)	21 (2.4)	

Supplemental Table V. Bleeding events reported in the included studies

[*] p value not reported. † $p = 0.041$. ‡ $p = 0.08$.

Supplemental Figures

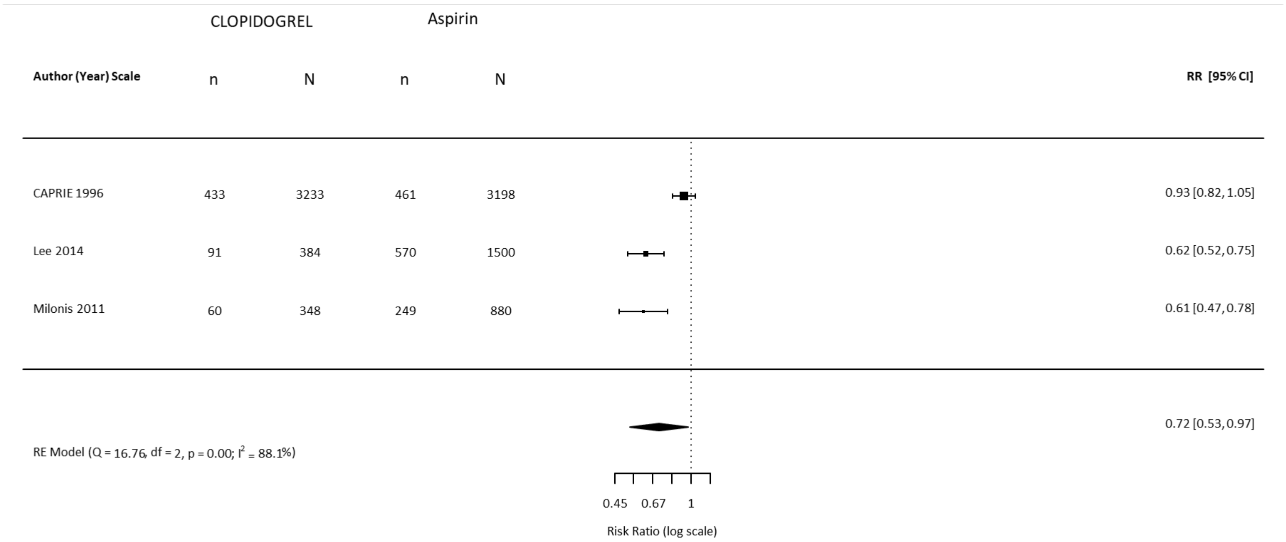

Supplemental Figure I. Sensitivity analysis for MACCE using the composite outcome of ischemic or hemorrhagic stroke or MI (not including death) from Lee et al. (2014).

Supplemental References

1. CAPRIE Steering Committee. A randomised, blinded, trial of clopidogrel versus aspirin in patients at risk of ischaemic events (CAPRIE). *Lancet* 1996; 348: 1329-1339. 10.1016/s0140-6736%2896%2909457-3.
2. Chi NF, Wen CP, Liu CH, et al. Comparison Between Aspirin and Clopidogrel in Secondary Stroke Prevention Based on Real-World Data. *J Am Heart Assoc 2018 Oct 02;7(19):e009856* 2018; 7: e009856-e009856. Journal Article. DOI: 10.1161/JAHA.118.009856.
3. Christiansen CB, Pallisgaard J, Gerds TA, et al. Comparison of antiplatelet regimens in secondary stroke prevention: a nationwide cohort study. *BMC Neurolology* 2015; 15: 225. 10.1186/s12883-015-0480-4 2015/11/04. DOI: 10.1186/s12883-015-0480-4.
4. Lee M, Wu YL, Saver JL, et al. Is clopidogrel better than aspirin following breakthrough strokes while on aspirin? A retrospective cohort study. *BMJ open* 2014; 4: e006672. 10.1136/bmjopen-2014-006672.
5. Milionis HJ, Gerotziafas G, Kostapanos MS, et al. Clopidogrel vs. aspirin treatment on admission improves 5-year survival after a first-ever acute ischemic stroke. data from the Athens Stroke Outcome Project. *Archives of Medical Research* 2011; 42: 443-450. 10.1016/j.arcmed.2011.09.001 2011/09/20. DOI: 10.1016/j.arcmed.2011.09.001.
